# Greatly increased occurrence of breast cancers in areas of mammographically dense tissue

**DOI:** 10.1186/bcr1260

**Published:** 2005-06-08

**Authors:** Giske Ursin, Linda Hovanessian-Larsen, Yuri R Parisky, Malcolm C Pike, Anna H Wu

**Affiliations:** 1 Department of Preventive Medicine, Keck School of Medicine, University of Southern California/Norris Comprehensive Cancer Center, Los Angeles, California, USA; 2 Department of Radiology, Keck School of Medicine, University of Southern California/Norris Comprehensive Cancer Center, Los Angeles, California, USA

## Abstract

**Introduction:**

Mammographic density is a strong, independent risk factor for breast cancer. A critical unanswered question is whether cancers tend to arise in mammographically dense tissue (i.e. are densities directly related to risk or are they simply a marker of risk). This question cannot be addressed by studying invasive tumors because they manifest as densities and cannot be confidently differentiated from the densities representing fibrous and glandular tissue. We addressed this question by studying ductal carcinoma *in situ *(DCIS), as revealed by microcalcifications.

**Method:**

We studied the cranio-caudal and the mediolateral-oblique mammograms of 28 breasts with a solitary DCIS lesion. Two experienced radiologists independently judged whether the DCIS occurred in a mammographically dense area, and determined the density of different areas of the mammograms.

**Results:**

It was not possible to determine whether the DCIS was or was not in a dense area for six of the tumors. Of the remaining 22 lesions, 21 occurred in dense tissue (test for difference from expected taken as the percentage of density of the 'mammographic quadrant' containing DCIS; *P *< 0.0001). A preponderance of DCIS (17 out of 28) occurred in the mammographic quadrant with the highest percentage density.

**Conclusion:**

DCIS occurs overwhelmingly in the mammographically dense areas of the breast, and pre-DCIS mammograms showed that this relationship was not brought about by the presence of the DCIS. This strongly suggests that some aspect of stromal tissue comprising the mammographically dense tissue directly influences the carcinogenic process in the local breast glandular tissue.

## Introduction

On a mammogram fat appears radiolucent or dark, whereas connective and epithelial tissue appear radiodense or white. The amount of mammographic density is a strong independent predictor of breast cancer risk [1-4]. For technical reasons, mammographic density has usually been expressed as mammographic percentage density (MPD; i.e. the ratio of the area of the breast that is dense to the total area of the breast on the mammogram). There is an approximately fivefold increased breast cancer risk in women with 60% or more MPD as compared with women with under 10% MPD, with a steady increase in risk with increasing MPD. Studies in which MPD is used directly have found very similar effects [5-8].

The biological basis for this increased risk for breast cancer associated with increased mammographic densities is not understood, and the detailed nature of densities has not been studied extensively. A fundamental question that has yet to be answered is whether, within a particular breast, cancers tend to occur in dense areas or not. Invasive carcinoma is usually identified as a spiculated dense mass on the mammogram, and even an expert mammographer is frequently unable to distinguish this mass from normal dense breast tissue, making it difficult, and often impossible, to decide whether the tumor arose in a dense area. We used mammograms obtained from patients with ductal carcinoma *in situ *(DCIS), as evidenced mammographically by microcalcifications, to address this question. DCIS is a 'nonobligate but definite local precursor of invasive carcinoma' [9], which commonly manifests as calcifications on the mammogram and usually can easily be differentiated from dense breast tissue.

## Materials and methods

We retrospectively identified consecutive women diagnosed with DCIS at the Henrietta C Lee Breast Center at the USC/Norris Comprehensive Cancer Center, for whom diagnostic mammograms were available at the Breast Center. There were 31 such patients. The study protocol was approved by the Institutional Review Board of the University of Southern California School of Medicine; patient informed consent was not required for this retrospective study, with minimal risk to participant.

For each participant we obtained the mammogram(s) of the affected breast(s) at the time of the DCIS diagnosis, and whenever available we also obtained the most recent pre-DCIS mammogram(s). We eliminated four of these women from further consideration at this stage: one because the DCIS lesions occurred over a wide area; one because the radiologists could not decide on the precise location of the DCIS (see below); and two because the single DCIS lesion occurred in the subareolar area – an area that is uniformly dense. Of the remaining 27 women, one had a single DCIS lesion in both breasts and 26 had a single DCIS lesion in one breast. These 28 lesions comprise the subject of this report. The DCIS tumors ranged in size (length) from 0.5 cm to 9.3 cm, and all but four were less than 4 cm. Eleven tumors were grade 3, eight were grade 2, one was grade 1, and seven were of unknown grade.

For each affected breast, the cranio-caudal (CC) and mediolateral-oblique (MLO) mammographic views were studied. Based on these two mammograms for each DCIS lesion, the two expert mammography radiologists (LHL and YRP) independently coded the DCIS lesion as to whether it was in an area of mammographically dense tissue (yes/no/cannot determine).

The breast images were also divided equally into a lateral and medial part based on the CC image (CC-L and CC-M, respectively); similarly, the MLO view was divided into a superior and inferior part (MLO-S and MLO-I, respectively). The radiologists independently visually assessed the percentage of mammographically dense tissue for each of the four 'mammographic areas' (CC-L, CC-M, MLO-S and MLO-I). The MPD for the whole breast was taken as the average of these four values. The percentage density of the part of the breast ('mammographic quadrant') containing the DCIS lesion was estimated as the average of the two density assessments of the mammographic areas containing the lesion. For instance, if the DCIS lesion occurred in the upper outer clinical quadrant of the breast, then it would be observed in the superior MLO view and the lateral part of the CC view, and the percentage density of the DCIS-containing mammographic quadrant would then be the average density of the CC-L and MLO-S mammographic areas. The percentage density of the part of the breast not containing the DCIS lesion was taken as the average of the remaining two density assessments. It is possible that more optimal measures of the density of that part of the breast containing, and not containing, the DCIS could be made, but this simple averaging measure is sufficient for our purposes here and it is not clear how one would determine a better measure.

Each DCIS lesion was classified as being in dense tissue (score of 1) or in nondense tissue (score of 0) or not determined (scored as a missing value). The expected score for a lesion was taken to be the PMD of the part of the breast containing the DCIS as described above (this was done separately for the estimated percentage density, as recorded by the radiologists individually and as their average values). The total score for the lesions was compared with the total expected score using exact methods for combinations of independent binomial distributions with known expected values. We used the paired t-test method to test the significance of the differences between the densities in affected and nonaffected DCIS areas in a single breast. All *P *values are two sided.

## Results

Table 1 shows the results for our main question of interest, namely whether DCIS occurs preferentially in mammographically dense tissue. Of the 28 images, radiologists 1 and 2 agreed on the rating for 24 of them: 21 as being in dense tissue, one as being in nondense tissue, and two as being associated with a part of the mammogram where the lesion was too closely associated with both dense and nondense tissue to be able to make an assignment confidently. Of the remaining four lesions, radiologist 2 was not willing to make a call on three of them whereas radiologist 1 felt that the lesion was in dense tissue, and there was a single lesion in which the two radiologists disagreed (radiologist 1 called it in nondense tissue whereas radiologist 2 called it as being in dense tissue). Of the 22 informative lesions with agreement between radiologists, 21 were called as being in dense areas (observed 21; expected 10.75 based on the assessment by radiologist 1, 10.77 based on the assessment by radiologist 2, and 10.76 for their average; *P *< 0.0001 using any of the three expected values).

Table 2 shows the average density (for the two radiologists combined) and the number of DCIS lesions by mammographic area (CC-M, CC-L, MLO-S and MLO-I). Of the 28 lesions, 17 occurred in the lateral-superior mammographic quadrant, whereas only two occurred in the medial-inferior mammographic quadrant. This correlated strongly with the average percentage density in the different mammographic quadrants, which varied from 55.8% in the lateral-superior mammographic quadrant (i.e. average of densities in the CC-L and MLO-S) to 38.3% in the medial-inferior mammographic quadrant (i.e. average of densities in the CC-M and MLO-I). Not surprisingly, we also found significant differences in densities between the lateral (*n *= 21) and medial (*n *= 7) areas of the mammographic CC view (51.3% for CC-L versus 33.5% for CC-M), and between the superior (*n *= 22) and the inferior (*n *= 6) areas of the MLO view (47.6% for MLO-S versus 34.7% for MLO-I). These two mammographic views are highly correlated; for example, the correlation between the percentage densities of the CC-L area and the MLO-S area was 0.93.

We also considered tumor grade and tumor size in our analysis. We found no clear association between tumor grade and percentage density, and the results were essentially unchanged when we excluded the four women with tumors greater than 4 cm (data not shown).

## Discussion

The findings of this study show that DCIS occurs overwhelmingly in the areas of the breast that are mammographically dense. We identified previous mammograms showing no DCIS for 13 of the 21 mammograms with DCIS in a dense area; all 13 showed that the areas subsequently showing DCIS were clearly dense at the time of the earlier mammogram.

Our results further show that DCIS occurs in the part of the breast that has the highest percentage density on the mammogram, namely the lateral-superior mammographic view. Although the difference in densities between the lateral-superior and the medial-lower mammographic quadrants (55.8% versus 38.3%) is highly statistically significant (*P *< 0.0001), the magnitude of the difference does not mirror the difference in frequency of DCIS and suggests that the amount of mammographically dense tissue is not the only component of the breast that plays a key role in the carcinogenic process.

Mammographic density is a very strong risk factor for breast cancer. The findings reported here suggest that this is a direct causal connection between the dense tissue and the breast glandular tissue. One possible biological basis for this would be that breast glandular tissue is overwhelmingly concentrated in the (mammographically) dense areas of the breast. Although, in our experience, this is generally believed to be true, there are surprisingly few data on this, and a recent study [10] found no correlation between the amount of glandular tissue in a breast and the mammographic density of the breast. There is much evidence from studies in rodents of a complex interaction between breast stroma and epithelial tissue [11], and increased epithelial cell proliferation secondary to increased density could explain why mammographic density is associated with an increased risk for epithelial malignancy.

Mammographic densities are themselves changed by interventions that affect breast cancer risk. Selective estrogen receptor modulators reduce breast cancer risk and reduce densities [12-14], and estrogen–progestin replacement therapy increases breast cancer risk and increases densities [15,16]. The interaction between the stroma and the epithelium may therefore be two way.

## Conclusion

Much remains to be learned, but this study shows that it is the nature of the interaction between stromal and epithelial tissue that should be the major focus of breast cancer research.

## Abbreviations

CC = cranio-caudal; DCIS = ductal carcinoma *in situ*; MLO = mediolateral-oblique; MPD = mammographic percentage density.

## Competing interests

The author(s) declare that they have no competing interests.

## Authors' contributions

All authors participated in the design of the study. GU coordinated the study, drafted the manuscript and contributed to the statistical analysis. LHL and YRP analyzed the mammograms. MCP contributed to the statistical analysis. AHW helped draft the manuscript. AHW and MCP conceived of the study. All authors read and approved the final manuscript.

This work was supported by the USC/Norris Comprehensive Cancer Center Core Grant P30 CA14089 (MCP). The funding source had no role in this report.

## Figures and Tables

**Table 1:** Relation of DCIS lesion to dense tissue

		Radiologist 2		
		
		Dense	Nondense	Cannot determine
Radiologist 1	Dense	21	1	0
	Nondense	0	1	0
	Cannot determine	3	0	2

DCIS, ductal carcinoma *in situ*.

**Table 2:** Average mammographic density and location of DCIS by 'mammographic quadrant' of the breast

Mammographic quadrant			
		
CC	MLO	Density (%; mean ± SE)	Number of DCIS lesions
L	S	55.8 ± 4.2	17
M	S	47.9 ± 4.0	5
L	I	46.2 ± 4.0	4
M	I	38.3 ± 4.0	2

CC, cranio-caudal; DCIS, ductal carcinoma *in situ*; I, inferior; L, lateral; M, medial; MLO, mediolateral-oblique; S, superior; SE, standard error.
